# Understanding Fabrication Variability in Core‐Shell Soft Biomaterials Using Stochastic Artificial Intelligence

**DOI:** 10.1002/advs.202522389

**Published:** 2026-04-01

**Authors:** Maria Alexaki, Lília M. S. Dias, Raquel C. Gonçalves, Dinis O. Abranches, Albano N. Carneiro Neto, Rute A. S. Ferreira, Paulo S. B. André, João F. Mano, Mariana B. Oliveira

**Affiliations:** ^1^ CICECO—Aveiro Institute of Materials Department of Chemistry University of Aveiro Aveiro Portugal; ^2^ Department of Physics and CICECO—Aveiro Institute of Materials University of Aveiro Aveiro Portugal; ^3^ Department of Electrical and Computer Engineering and Instituto de Telecomunicações Instituto Superior Técnico Universidade De Lisboa Lisbon Portugal; ^4^ Department of Electrical and Computer Engineering Carnegie Mellon University Pittsburgh Pennsylvania USA

**Keywords:** Gaussian processes, hydrogels, soft biomaterials, stochastic machine learning, uncertainty

## Abstract

Approaches for the fabrication of biomaterials are currently numerous, with a wide diversity of available material precursors, chemistries, and processing technologies. Owing to the complex nature of the human body, biomaterials are targeted for applications with highly diverse performance demands. Traditional strategies based on trial and error have fallen short of predicting the ideal parameters required to produce adequate structures to meet these challenges. Although the design of experiments enables reducing experimental testing, it has failed to predict complex, multi‐factorial processing effects, including experimental variability. Despite being often overlooked, experimental variability is an important aspect in biomaterials, which are often processed from source materials with significant compositional variability (e.g., natural polymers), along with processing methodologies frequently undertaken under poorly controlled environmental conditions. Here, a machine learning approach based on Gaussian processes (GPs) is developed to identify patterns and correlations between fabrication conditions and material properties. Flexible soft membrane‐based tubular materials obtained by polyelectrolyte complexation are used as a model biomaterial characterized by multi‐parametric design inputs. Using GPs, the effects of processing parameters on the magnitude and variability of key properties like permeability, porosity, thickness, opacity, and swelling ratio are quantified. This approach is expected to enable more reliable and predictable biomaterial fabrication.

## Graphical abstract

Fabrication‐induced variability remains a fundamental limitation in the scalable design of soft biomaterials. In this work, a stochastic machine learning approach based on Gaussian processes modeling is employed to establish quantitative links between biofabrication parameters, material properties, and their intrinsic variability. Core–shell hydrogel tubes serve as a model biofabrication system, enabling advancing variability‐aware strategies for reliable biomaterial manufacturing.

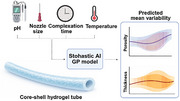

## 1 Introduction

Exhaustive research has contributed to the collection of extensive datasets on biomaterial properties and their processing techniques. From initial trial‐and‐error approaches, the field has evolved by establishing relationships between properties and composition, processing techniques, or molecular structure, using high‐throughput methodologies based on design of experiments methodologies and analyzed using multivariate statistical approaches [1]. More recently, machine learning (ML) has been explored as a tool to uncover relevant information and insights on materials properties, even from unstructured empirical results [2]. The growth of the field is striking, with the potential of ML for the design and discovery of new functional materials fully recognized by several communities [3, 4, 5, 6, 7]. Although this trend is also observed in the biomaterials field, the effective application of ML is still scarce, with most publications in the field comprising opinion and literature reviews and perspectives [8, 9, 10, 11, 12], and few ML approaches focused on a reduced number of biomaterial classes [13, 14, 15]. Additionally, predicted outputs commonly address biological activity, and the established relationships often focus on the molecular structure of raw precursors (e.g., monomers, polymers, ceramic precursor compositions), or surface/bulk properties of materials, including wettability and elastic modulus [16, 17, 18]. While the interactions of cells with the surface of materials determine the functionality of most hard materials (e.g., ceramics and glasses), the functionality of soft materials – usually of polymeric nature and with high hydration properties – typically depends on more complex features that include 3D disposition of pores, overall porosity, and dynamic properties such as swelling kinetics, degradation, and viscoelasticity. Those intrinsic physical features of hydrogels and soft matter‐based analogues have not been addressed using ML approaches, and the exploration of their variability within ML modelling has been, so far, overlooked.

Core‐shell fibers assembled at the interface of all‐aqueous systems are here used as materials with a non‐continuous architecture – comprising a fully liquid core and a hydrogel membrane – [19], to study the dependency of a range of processing parameters on the typical features of those compartmental soft systems. Here, we focused on a design space directed for common requirements of cell encapsulation and bioprinting applications. Those require carefully constrained processing conditions to preserve cell viability. For this reason, the experimental design space in this study was deliberately restricted to cytocompatible and application‐relevant regimes, including near‐physiological pH values, room temperature to physiological processing temperatures ranges, practical nozzle sizes, and short complexation times commonly used in cell‐laden hydrogel systems. Rather than exploring extreme parameter ranges that may induce cytotoxicity or compromise biological relevance, the study focuses on small, controlled variations around standard operating conditions typical of cell encapsulation workflows. This targeted design space enables the identification of subtle yet meaningful process‐structure relationships while ensuring that the resulting models remain directly applicable to realistic biofabrication scenarios. A detailed examination of how different processing parameters affect tube properties, including membrane formation kinetics and functional outputs with practical utility, including mass transport kinetics, is still lacking. More importantly, defining adequate processing variables that could enable building reliable predictive tools is crucial to accelerating the design and production of those materials with targeted performance, for example, for bioengineering and drug delivery applications.

**FIGURE 1:**
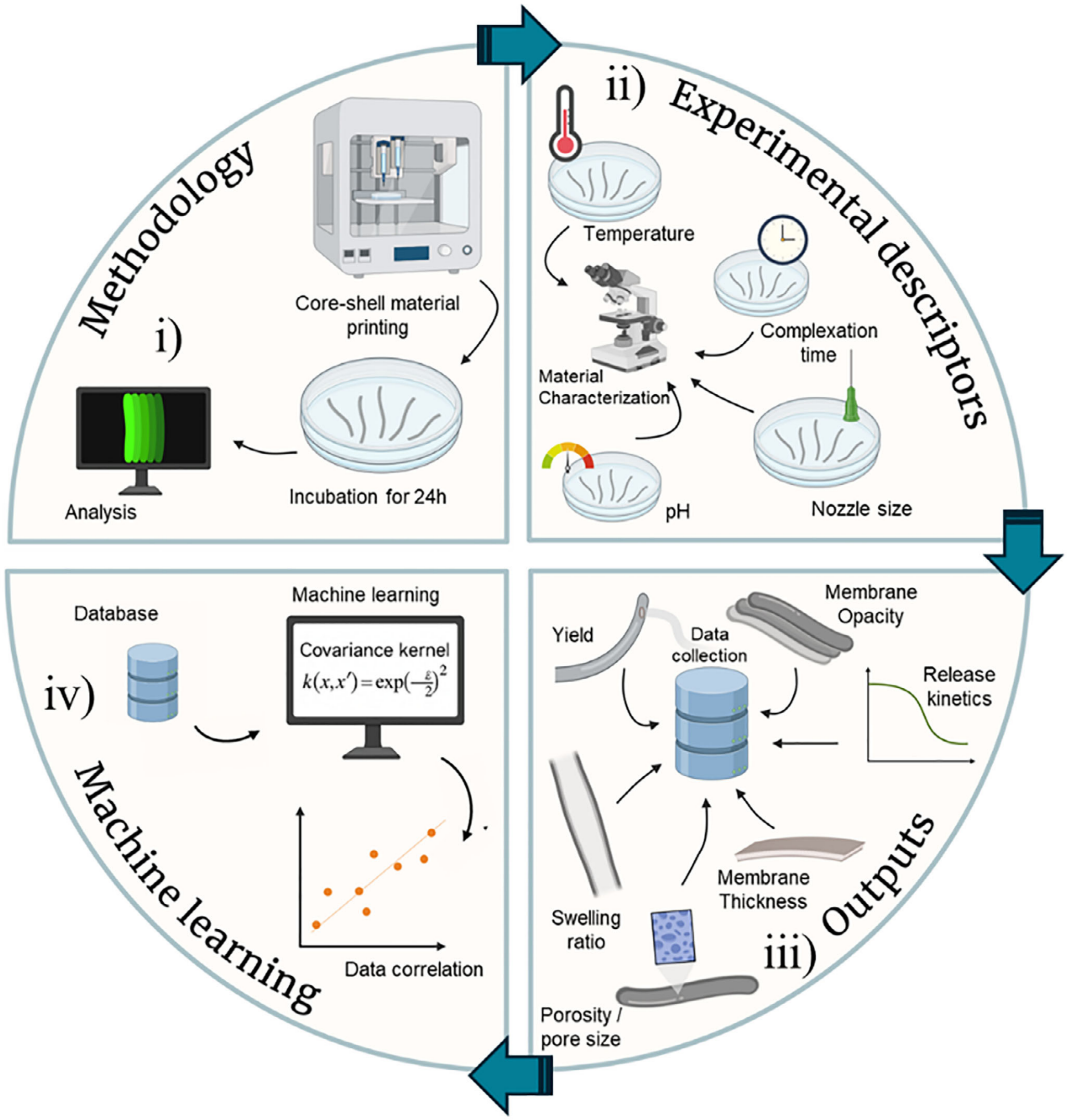
Schematic representation of the workflow adopted in our study, starting with (i) the establishment of a methodology to prepare biomaterial samples in large scale, followed by (ii) the design of a combinatorial experiment that led to the (iii) retrieval of data (outputs), which finally enabled the (iv) creation of a new dataset used to train a ML algorithm and predict material properties.

The results of a systematic study on the processing of core‐shell polymeric tubes (Figure 1) are analyzed, and immediate observations are discussed. Measurement data were compiled into a comprehensive database that was used to train Gaussian processes (GPs) for predictive modelling. GPs are a class of stochastic ML models that are efficient at describing small and scarce datasets. Owing to their stochastic nature, GPs handle experimental uncertainty and noise natively, treating output variables as random variables that follow a normal probability distribution, meaning that GP predictions are always associated with a standard deviation. Thus, for any given prediction, GPs provide an estimate of their uncertainty, which encompasses both the epistemic uncertainty of the model as well as the anticipated experimental variability of the property [20]. GPs are also non‐parametric models, which facilitates their training by removing the need for extensive hyperparameter tuning, in contrast with other ML models such as artificial neural networks. The capability of GPs to predict the properties of the processed tube‐shaped membranes and their intrinsic experimental variability was assessed. It is important to emphasize that the motivation for employing GPs in this work is not solely to maximize pointwise predictive accuracy, despite their compatibility with small and scarce datasets, but rather intrinsic uncertainty estimates alongside model predictions. In contrast, commonly used machine learning models such as random forests, gradient boosting, and neural networks are not stochastic and do not natively provide predictive uncertainty estimates without additional modeling assumptions or techniques. Accordingly, the value of GPs in this work lies primarily in their ability to quantify uncertainty, which is central to their usefulness in biofabrication applications. To the best of our knowledge, this is a novel approach that introduces advanced ML to analyze hydrogel biomaterials' properties and variability. It demonstrates how artificial intelligence (AI) can act as a powerful tool for material scientists, enabling a more efficient design and production of biomaterials tailored for targeted performance.

## 2 Results and Discussion

Figure 1 shows a schematic representation of the employed workflow, ranging from (i) biomaterial preparation and (ii) combinatorial experimentations to the acquisition of (iii) biomaterial characterization data that was included in a database for (iv) ML processing.

### 2.1 Materials Characterization

**FIGURE 2:**
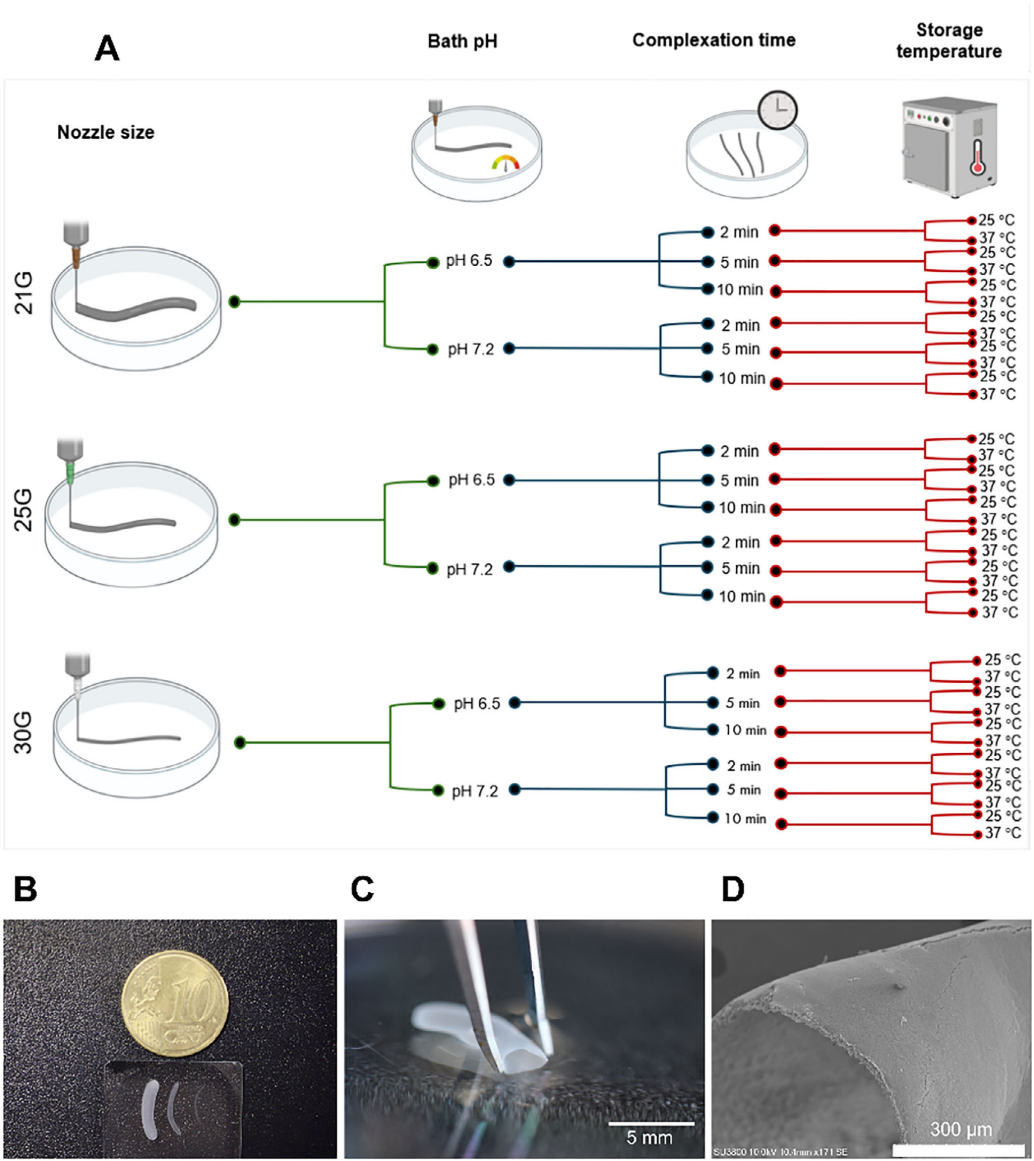
(A) Representation of the conditions tested in the experimental study. (B) Fiber samples processed with different nozzle sizes. (C) Picture of one fiber with a trimmed end, showcasing the hollow/liquid‐core features of the materials, also validated by scanning electron microscopy (SEM) (D).

The production of core‐shell fiber‐shaped materials through the interfacial complexation of two oppositely charged polyelectrolytes – epsilon‐poly‐L‐lysine (EPL) and alginate (ALG) – at the interface of a prototypic aqueous two‐phase system was here adapted to a fully automated 3D printing protocol, and an array of conditions was processed (Figure 1). Systematically varied parameters included the (i) pH of solutions; (ii) diameter of the nozzle used for extrusion; (iii) complexation time; and (iv) effect of storage temperature, according to the ranges shown in Figure 2A. Representative images of fibers processed using different nozzles were acquired (Figure 2B), and the presence of a hollow/liquid core on the structures was confirmed by optical and electronic microscopies (Figure 2C,D). Membranes were then characterized considering the following properties: yield of stable membranes obtained after washing; membrane estimated thickness and outer diameter; pore size, and porosity; swelling ratio; optical densification during complexation; and permeability to high molecular weight molecules.

**FIGURE 3:**
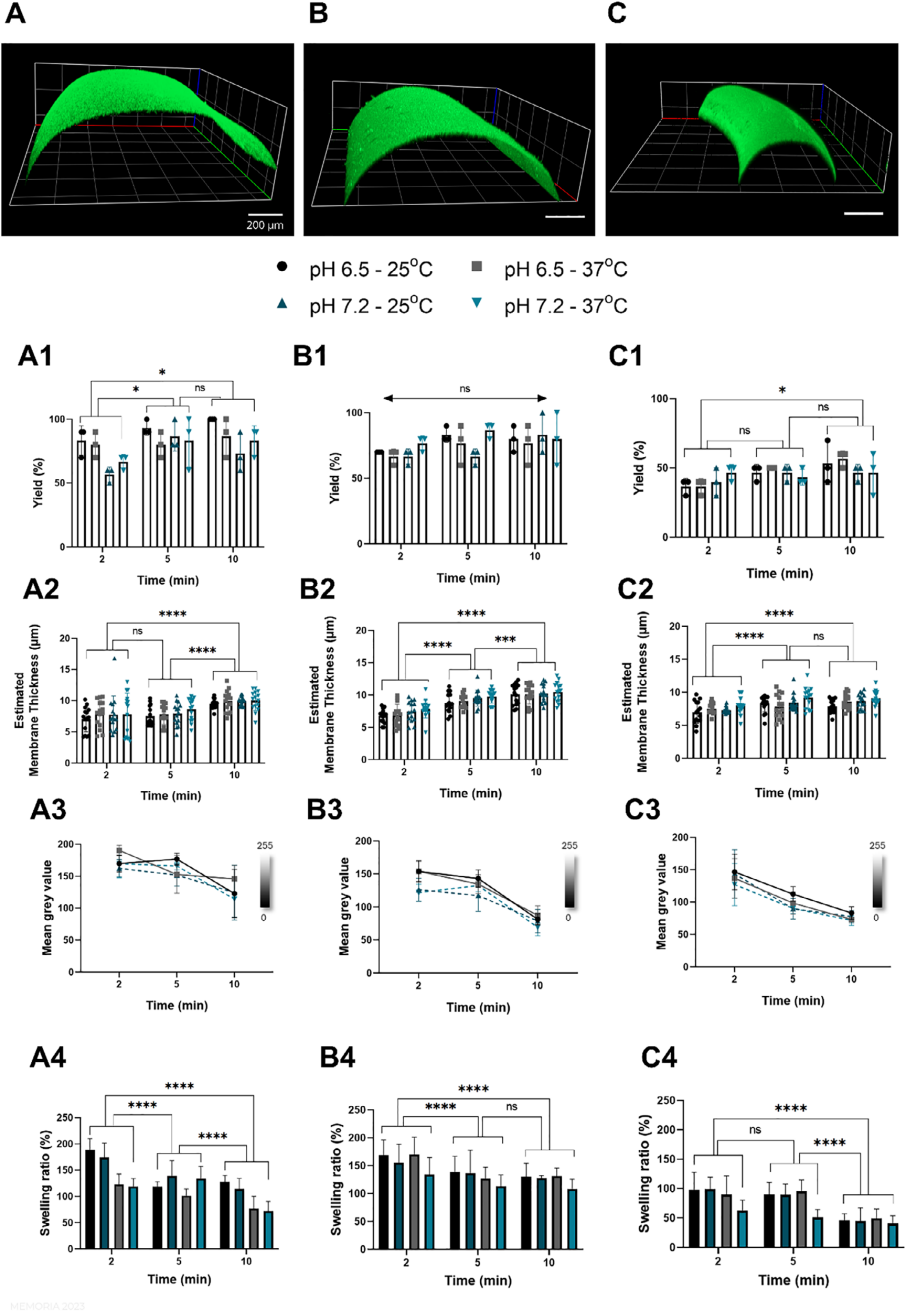
Characterization of fibers prepared using different processing nozzle sizes – (A) 22G, (B) 25G, and (C) 30G – with confocal microscopy images of FITC‐labeled fibers with their thickness evidenced. (A1–C1) Yield of stable fibers produced in an experimental run, with a total of 10 processed fibers. (A2–C2) Measured membrane thickness. (A3–C3) Variation of the mean grey value of fibers during complexation time. (A4–C4) Swelling of fibers after 24 h of immersion in PBS. Statistically significant differences between groups were assessed using two‐way ANOVA. ^*^, ^***^, and ^***^ indicate *p*<0.05, 0.005, and 0.001, respectively.

For the characterization of approximate thickness, pore size, and porosity, the use of fluorescent‐labeled fibers was performed using confocal microscopy. As expected, the diameter of the tubes varied with nozzle size. Specifically, the use of a 21G nozzle produced tubes with an outer diameter of approximately 2 mm, while the use of a 25G nozzle led to a reduction of diameters to approximately 1 mm. Further reduction in nozzle size to 30G led to tubes with a smaller diameter of approximately 0.5 mm (Figure 3A–C).

The effect of different post‐complexation treatments of the aqueous interface‐assembled tubes has not been studied before. After tube assembly and washing, samples were incubated overnight either at room temperature (∼25°C) or at 37°C. This step was essential to understand the impact of post‐processing storage temperature on material properties, especially for samples stored at 37°C, corresponding to intracorporeal temperature and, consequently, commonly used for in vitro cell culture. Given that the optimization of fiber processing protocols to produce continuous fibers was one of the most challenging aspects while optimizing interface‐assembled EPL‐alginate tubes [19], we evaluated the ability of fibers to keep a stable morphology, without visible rupture sites (here, addressed as yield), by measuring the ratio of stable continuous fibers to the total number of produced objects after 24 h of incubation in a physiological‐mimetic buffer (Figure 3A1–C1). Overall, longer complexation times improved fiber stability, while shorter periods (e.g., 2 min) led to the formation of fragile membranes with a tendency for rupture. Two pH values (6.5 and 7.2) were selected for fabrication to ensure mild processing conditions that can be readily translated to applications where cytocompatibility is critical. The reduction from the near‐physiological pH of 7.2 to 6.5 was designed to assess whether a subtle decrease in the pH of the printing bath could measurably influence material properties. EPL behaves as a weak polyelectrolyte and exhibits a gradual response to pH variations around its pK_a_. As shown by its titration curve with a NaOH solution (Figure S12), the degree of protonation of EPL is slightly higher at pH 6.5 than at pH 7.2. Consequently, EPL was expected to display increased reactivity at pH 6.5, which may enhance its interaction with alginate carboxylic groups. Nonetheless, variations in the pH of the complexation/printing bath and further storage temperature showed minimal effects on the yield of stable fibers. On its turn, nozzle size showed an important role in the achieved rates of stable and continuous retrieved fibers after processing: larger nozzles—including 21 and 25G—lead to a higher yield of stable fibers (in general, > 60% of stable fibers). The smallest nozzle size – 30G—led to a significant drop in the fiber stability yield, which dropped to below 50% (Figure 3C1). This reduction is likely due to the increased shear forces associated with smaller nozzles during printing. The fine‐tuning of printing conditions using small‐sized nozzles may enable improving the successful retrieval of stable fibers with small dimensions.

Interestingly, nozzle size had an overall minimal impact on the membrane thickness of fibers (Figure 3A2–C2), showcasing residual variations in the interfacial membrane across varying diameters. In contrast, and as expected, complexation time significantly impacted the thickness of the membrane, with increasing complexation times leading to the formation of thicker membranes, following a close‐to‐linear variation within the same processing parameters. Of note, the post‐processing storage at different temperatures and the use of printing bath at different pH values showed negligible effects on the membranes’ thickness, which did not reflect statistical significance. The opacity of the membranes was measured during complexation to, in combination with thickness measurements, provide a deeper insight into membrane growth and possible densification. As expected, increasing complexation times led to the formation of fibers with overall increasing opacity. Fibers prepared with nozzles changing from 30 to 21G led to increasingly transparent membranes (Figure 3A3–C3). This suggests that, while thickness remained in general constant for fixed complexation times, an increase in opacity with decreasing nozzle sizes may be related to a higher membrane densification at the interface for small diameter constructs. While alginate–dextran mixtures were extruded at 60 kPa when using larger nozzle diameters, a threefold higher pressure was required for extrusion through the 30G nozzle. In addition, the printing speed was adjusted across conditions, with the 21G nozzle printed at a 2.6‐fold higher speed than the 30G nozzle. Such variations in printing parameters may influence the properties of the resulting materials through several mechanisms [21], including possible enhanced polymer chain alignment during extrusion, localized viscosity changes induced by higher shear or extrusion rates, or modulation of solvent exchange kinetics at the material–bath interface, which could ultimately lead to accelerated interfacial reactions. We also hypothesized that the storage of tubes after production, either at 25°C or 37°C, could impact the rearrangement of the complexed membrane, as well as the processing pH of the EPL bath. Overall, neither the pH of the printing bath nor the storage temperature for tubes led to consistent variations in the tube transparency after 24 h of incubation in phosphate‐buffered saline (PBS).

The swelling of tubes upon immersion in PBS at different temperatures was also assessed. Structures with shorter complexation times and thinner membranes exhibited higher swelling ratios compared to those with longer complexation times. Also, as expected, increasing complexation times led to lower swelling ratios, as previously reported [19]. Interestingly, the overall swelling ratio of tubes decreased with decreasing fiber diameter (Figure 3A4–C4). Although all structures presented similar membrane thicknesses for fixed processing parameters other than varying nozzle size (Figure 3A1–C1), opacity detected for tubes with larger diameters was lower, suggesting the less dense feature of their membranes, which may explain their higher tendency to absorb and expand with water. Following a similar trend to previously assessed fiber features, EPL bath pH and storage temperature did not show a consistent role in the swelling ratio of the structures.

**FIGURE 4:**
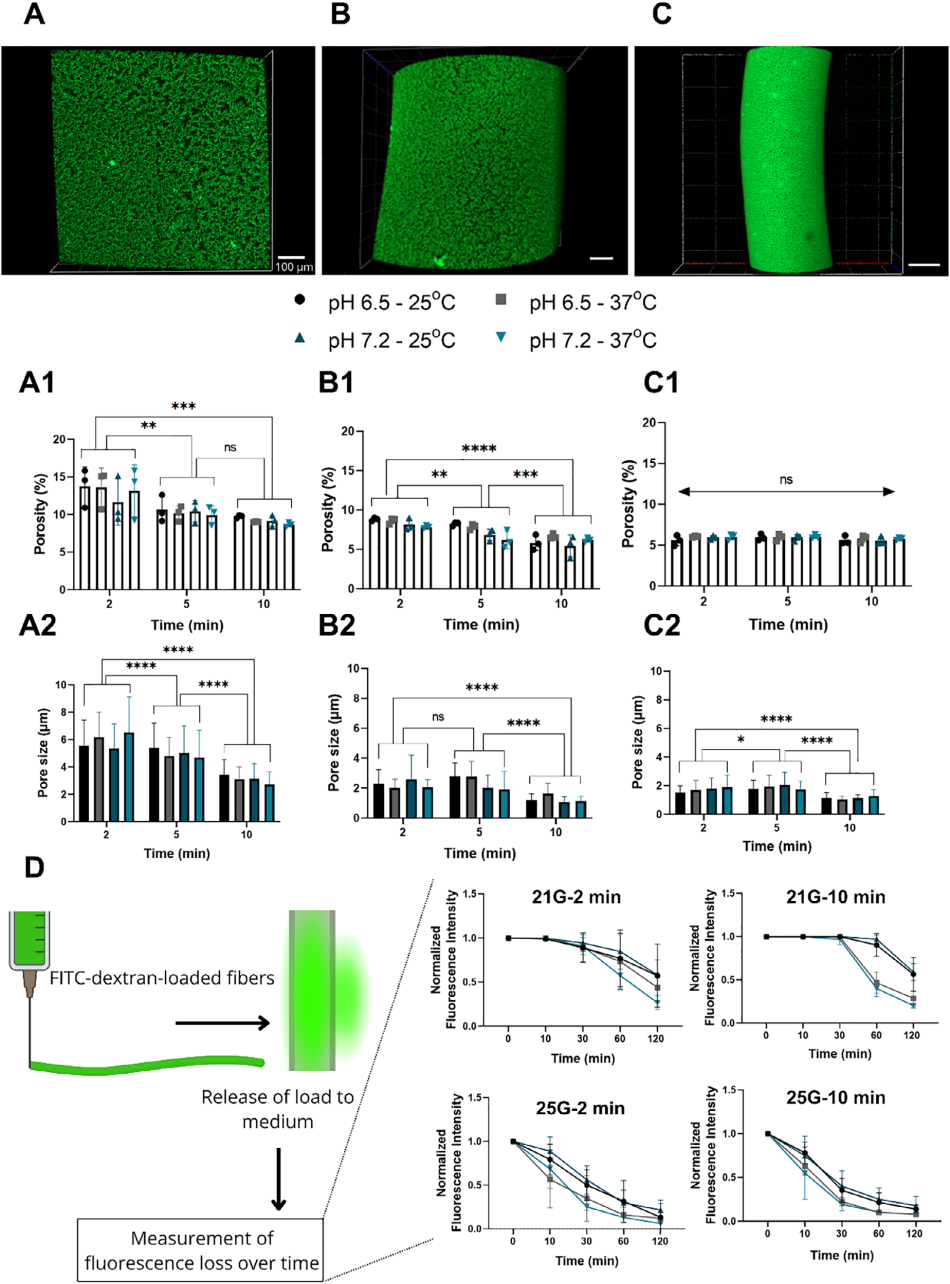
Characterization of the membrane surface for fibers prepared with (A) 22G, (B) 25G, and (C) 30G extrusion nozzles. (A1–C1) Characterization of the porosity of the membranes of different fibers (*n* = 3 fibers). (A2–C2) Pore size characterization, with values presented for 20 pores/fiber (*n* = 3 fibers). (D) Characterization of the release profile of 2000 kDa dextran‐FITC loaded during fiber manufacturing. Statistically significant differences between groups were assessed using two‐way ANOVA. ^*^, ^**^, ^***^, and ^***^ indicate *p* < 0.05, 0.01, 0.005, and 0.001, respectively.

The porosity and pore size of the membranes were assessed after 24 h of incubation in PBS. Similar to the previously discussed properties, nozzle size and complexation time were the main processing factors whose variations correlated with tendencies in both pore size and porosity of the membranes. Membrane porosity decreased in an apparently close‐to‐linear manner with increasing complexation times, both for samples prepared with 21 and 25G nozzles (Figure 4A–C; 4A1–C1). Interestingly, this tendency was not observed for tubes processed with 30G needles, suggesting that small‐diameter tubes reach an equilibrium in their organization faster than their larger diameter counterparts. Also, for pore size, nozzle size had a relevant effect, with the highest nozzle size – 21G—leading to pore sizes in the range of 6 µm to 3 µm, with increasing complexation time from 2 to 10 min, respectively. For the 25G nozzle, pore sizes ranged from average values of 2.5 µm to 1 µm, also with pore size decreasing as the complexation time increased (Figure 4A2–C2). A similar pattern was observed with the use of the 30G nozzle, in which pore sizes ranged from 2 µm to 1 µm. The lack of consistent variations with bath pH and temperature corroborates that nozzle size and complexation time are the primary drivers of membrane morphology in the reported system.

The permeability of interface‐complexed tubes of alginate and EPL was previously reported for a single formulation, prepared with a 25G needle [19]. The diffusion of molecules up to 150 kDa was observed, with 70% of the total cargo released after 60 min of incubation in PBS at room temperature [19]. Here, we assessed the ability of the processed membranes to enable the permeation of molecules with an overall size in the nanometric range, with ca. 50 nm [23] (Figure 4D). Two nozzle sizes – 21 and 25G—were tested; fibers prepared using the 30G needles were excluded from the analysis due to their low stability yield. A time of complexation of 2 min was tested, as this was the condition previously associated with the highest cell viability [19], so the retrieved data could provide valuable information about the exchange of biopharmaceuticals or cell‐derived secreted molecules and vesicles. An additional complexation time of 10 min was tested to understand the possible correlation of higher membrane estimated thickness with the release profile of large molecules. Overall, all processed fibers enabled the passage of the macromolecule through the membrane, although at different diffusion rates. For fibers prepared using 21G needles, increasing complexation time led to slower release rates, with retention times ranging from 10 to 30 min before detectable release of the molecules, for 2‐ and 10‐min complexation, respectively. Despite the apparent higher membrane densification observed for smaller diameter fibers, our findings also showed that reducing the material size by using smaller needles (25G) resulted in significantly faster release rates compared to larger needles (21G). Considering the membranes prepared in both conditions showed porosities and pore sizes in the same order of magnitude, with tubes prepared with 25G showing threefold smaller pore size, the slower release kinetics observed for larger membranes may be ascribed to architectonic differences in the porous inner part of the membrane, which probably lead to a more efficient entrapment of the large dextran macromolecules. Also, for fibers prepared with 25G needles, complexation time did not play any detectable role in the overall release kinetics of 2000 kDa dextran from the membrane. As a general tendency, temperature played a key role, with experiments performed at 37°C leading to accelerated release rates, most noticeable for fibers prepared with 21G needles. This may be attributed to the higher kinetic energy at higher temperatures, leading to probably faster molecular movement.

### 2.2 Gaussian Processes Analysis

#### 2.2.1 Gaussian Processes for Prediction of Physical Properties

In the previous section, a comprehensive experimental database of biomaterial properties was generated considering a significant range of biofabrication process parameters (see Figure 2A). Reframing this dataset within the context of conventional ML terminology, it comprises four features (ML model inputs, as depicted in Figure 2A) and eight labels (ML model outputs). Features include pH (6.5 or 7.2), storage temperature (25 or 37°C), nozzle size (21G, 25G, or 30G), and complexation time (2 min, 5 min, or 10 min), while labels consist of yield of stable membranes obtained after washing (108 samples), swelling ratio (505 samples), thickness (540 samples), opacity (324 samples), porosity (108 samples), pore size (2160 samples), release constant (48 samples), and fluorescence at plateau (144 samples).

The physical properties mentioned in the previous paragraph were measured using multiple experimental replicates for each biomaterial sample. In accordance with standard practices in the field, these replicates are typically averaged to produce a single representative mean value, accompanied by a standard deviation, which is then used to train ML models. However, owing to their stochastic nature, it is possible to train GP models using individual replicate measurements rather than aggregated data, an approach seldom taken in the literature. Considering the exceedingly large variability of the data measured and reported in this work, as explained in the previous section and as is typical of biofabrication processes, namely those of hydrogels, all individual replicate values were retained and used to train the GP models.

**FIGURE 5:**
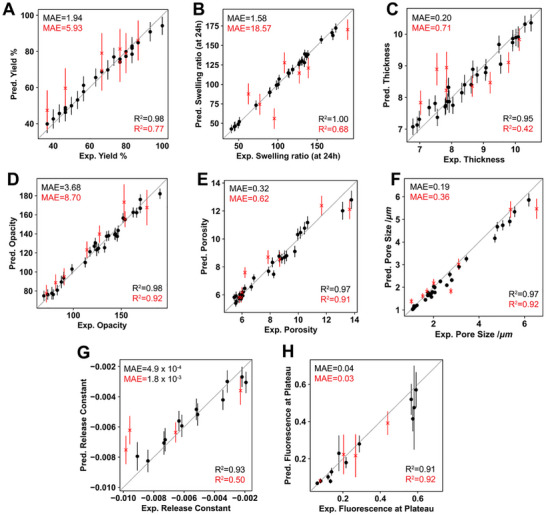
GP performance, in terms of predicted versus experimental property, for the training (black) and testing (red) datasets of each label (i.e., biomaterial property) studied in this work, namely yield of stable membranes obtained after washing (A), swelling ratio (B), estimated thickness (C), opacity (D), porosity (E), pore size (F), release constant (G), and fluorescence at plateau (H). GPs were trained using each individual experimental replicate for each property and experimental condition. GP‐predicted uncertainties are provided as error bars. The mean absolute errors (MAE) and coefficient of determination (R2) are displayed. The dashed line represents y = x.

For each target label (i.e., biomaterial property, denoted as *Y*), a GP model of the following form was obtained:

(1) $$\mu_{Y},s_{Y}=GP\left(x_{1},x_{2},x_{3},x_{4}\right)$$

where *x*1 − *x*4 are the four features described above (pH, temperature, nozzle size, and complexation time), while µ_*Y*_ and *s_Y_* are, respectively, the GP‐predicted mean, corresponding to the expected value of *Y*, and the predicted standard deviation, which reflects the uncertainty of the model in that prediction. As stated above, GPs were fitted to all measured data replicates. Thus, unlike what is usually done in the literature, the understanding of the intrinsic variability of each data point was left to the stochastic capabilities of the GPs instead of computing, by hand, a mean and standard deviation for each set of replicates. Furthermore, to mitigate the risk of overfitting and assess the generalizability of the models, each dataset was split into training (80%) and testing (20%) sets using stratified sampling. This method ensures that the distribution of data points in both sets reflects the variability present in the full dataset. The resulting distributions for each biomaterial property are reported in Figure S2. Note that the testing set data represent novel experimental biofabrication conditions never seen by the model during training. Thus, the testing set performance metrics effectively evaluate the capability of GPs to generalize to unseen conditions and novel material structures and properties. Following standard ML practices, the normalization strategy for each label was selected based on its distribution profile. Specifically, standardization was applied to labels exhibiting approximately normal distributions, while log‐standardization was used for those displaying log‐normal characteristics. Due to the low diversity of features in the database, feature normalization was only carried out when overfitting of the training set (testing coefficient of determination, R^2^ = 1) was observed. A list of the normalization methods applied in this work for each dataset, along with the corresponding label value ranges, is provided in Table S1. The performances of each trained GP model, incorporating the chosen normalization techniques, are depicted in Figure 5. This includes both the predicted means and the associated uncertainties, which reflect the GP‐predicted experimental variability for each biomaterial property.

The results depicted in Figure 5 reveal several interesting phenomena. First, the performance of the GP models on the training sets is consistently high across all target properties, with coefficients of determination exceeding R^2^ = 0.90 in every case. This holds true even for properties characterized by substantial GP‐predicted uncertainties, such as porosity and fluorescence at the plateau. However, the generalization performance, which is evaluated using the testing sets and assesses the capacity of the model to make reliable predictions on unseen data, varies considerably depending on the specific biomaterial property. For properties such as opacity, porosity, pore size, and fluorescence at plateau, the GP models exhibit excellent predictive performance, with testing R^2^ exceeding 0.90. In contrast, the models perform less robustly for other properties, ranging from a relatively low testing R^2^ of 0.42 for membrane thickness to a moderate R^2^ of 0.77 for yield.

With regard to GP‐predicted uncertainties, it is interesting to note that the magnitude of the error bars in Figure 5 is consistent with the predictive performance of the model. In other words, GPs are able to anticipate their own deviations from experimental results, providing information on their expected reliability. For instance, error bars for the testing set of yield (R^2^ = 0.77) are considerably larger than those for the testing set of pore size (R^2^ = 0.92), indicating that the latter predictions are more trustworthy than the former.

So far, the results discussed highlight the capability of GPs to predict complex biomaterial properties, even under experimental biofabrication conditions never seen before by the model (testing set). Notably, the models account for the typical variability inherent to polymeric hydrogel‐like structures, demonstrating their robustness in handling experimental noise, as seen in the size of the GP‐predicted error bars for each case. Despite these strengths, the GP models exhibited limited predictive accuracy for membrane thickness and release constants. This is most likely connected to severe experimental variability and uncertainty, as will be discussed below. Note that the release studies of dextran molecules were performed using completely independent samples, prepared on different days and often from different solution baths. Additionally, this property is dependent on time and external conditions, including shaking and temperature stability, which were not treated as inputs (features) to the GP models.

#### 2.2.2 Gaussian Processes for the Prediction of Experimental Variability

**FIGURE 6:**
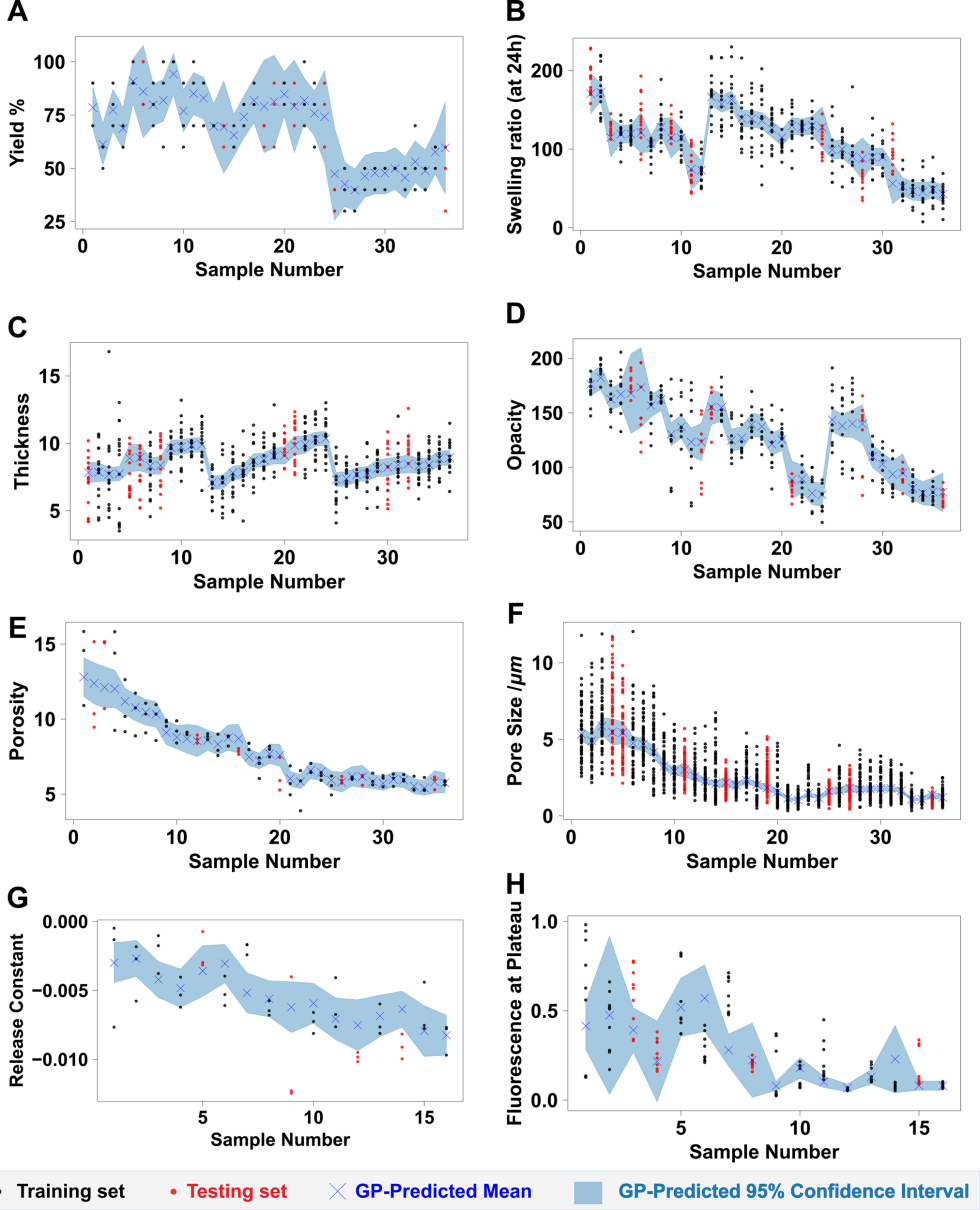
Individual experimental replicate measurements for each label (i.e., biomaterial property) used in this work to train GP models, namely yield of stable membranes obtained after washing (A), swelling ratio (B), thickness (C), opacity (D), porosity (E), pore size (F), release constant (G), and fluorescence at plateau (H). GP‐predicted means and 95% confidence intervals are also included. GPs were trained using each individual experimental replicate for each property and experimental condition.

To better understand the results discussed above and emphasize the significance of employing ML models capable of capturing experimental uncertainty and variability, Figure 6 presents the GP‐predicted properties for each independent biomaterial sample. These predictions are superimposed on the corresponding experimental replicated measurements to facilitate direct comparison. Additionally, 95% confidence intervals, obtained from the GP‐predicted standard deviations as defined in Equation (1), are included to illustrate the range of model uncertainty. This alternative data visualization underscores the ability of GPs not only to provide point estimates but also to quantify the reliability of their predictions, which is particularly valuable when modeling systems characterized by high experimental noise and variability.

Figure 6 provides valuable insights that help interpret the behavior of the GP models reported so far. It is clear that several properties, namely swelling ratio, membrane thickness, and release constant, are characterized by substantial experimental variability, as can be seen from the scatter of the experimental data points in Figure 6. These are the cases where GP performance on the testing set is less reliable. However, despite comparable levels of experimental variability for pore size, the corresponding GP models demonstrate markedly better predictive performance. It is important to note that this study intentionally focuses on response variables that exhibit substantial experimental variability, even under tightly controlled conditions, to evaluate the usefulness of GP models in such settings. The reduced predictive performance observed for some properties, such as membrane thickness and swelling, likely reflects the influence of additional, unmeasured experimental factors, as evidenced by their substantial intrasample variability (Figure 6). Notably, despite this variability, the GP models maintain high training performance while appropriately expressing increased predictive uncertainty for test data (Figure 5), with test set metrics being negatively influenced by a small number of outliers. Specifically, the model for pore size achieves a high coefficient of determination on the test set (R^2^ = 0.92), whereas, for example, the model for membrane thickness performs significantly worse (R^2^ = 0.42). At first instance, this difference may simply be attributed to disparities in the size of the training datasets (1680 data points for pore size vs. 420 for membrane thickness). However, this discrepancy can also be attributed to differences in the nature of variability present in the data. While both membrane thickness and porosity exhibit high intra‐sample variability (Figure 6), pore size shows pronounced inter‐sample variability, reflected in distinct mean values across different biofabrication conditions. This greater inter‐sample differentiation enables the GP model to identify more meaningful patterns and correlations between the input features and the target property.

These findings highlight an important consideration: when a property shows minimal inter‐sample variability, even sophisticated ML models may struggle to achieve high predictive accuracy, regardless of the approach used. In such cases, the inherent lack of distinguishable patterns across the dataset limits the ability of the model to generalize, suggesting that there are process parameters/features that are not being considered but have a significant impact on the outcome of the process. It is interesting to note that the achievement of low variability between processing conditions, namely the ones enabling the processing of biomaterials at different scale lengths, is usually aimed for by researchers in the field. For example, the achievement of similar pore size, porosity, and thickness in materials processed using different nozzle sizes – and therefore showing different diameters – is considered evidence of the versatility of the system. This consistency shows that the process can be scaled up or down without affecting the material's key properties, which is a valuable feature in biomaterials research.

Another noteworthy observation from the results presented in Figure 6 is the apparent overconfidence of the GP models for certain properties. For instance, while the GP model for swelling ratio achieved a relatively low testing set coefficient of determination (R^2^ = 0.68), the corresponding 95% confidence intervals are notably narrow. This suggests that the model is overly confident in its predictions, despite its limited predictive accuracy. This is a potential indication of inconsistencies or underlying issues within the experimental data that the GP is unable to fully capture. In contrast to this overconfidence, the GP model for pore size demonstrates a more appropriate alignment between prediction accuracy and uncertainty estimation. Despite the substantial experimental variability associated with this property, the model produces narrow confidence intervals. This behavior again underscores the advantages of stochastic ML in not only generating point predictions but also in quantifying uncertainty when the underlying data structure allows for reliable pattern recognition.

## 3 Conclusions

In this study, we report on the preparation of a *de novo* database using core‐shell tubes prepared by interfacial complexation at an all‐aqueous interface as a complex biomaterial model. We characterized previously unexplored processing parameters and output measurements, providing insight into working ranges and limits of the system. We showed that GP modeling was adequate to predict material properties based on experimental data with high efficiency, achieving coefficients of determination R^2^∼0.9 for key outputs such as opacity, porosity, and pore size. The model was designed using only four input features, highlighting the ability of the GP algorithm to identify and predict complex relationships even in datasets with limited size and variability. We delved into an unexplored aspect in ML studies for biomaterials: the ability of those models to predict the intrinsic variability of the systems. While some classes of materials are processed in highly controlled equipment, with well‐characterized pressure and thermal parameters and losses, hydrogels are typically processed in comparatively less defined conditions (e.g., room temperature) using multistep procedures that may introduce cumulative errors in the preparation of different samples and batches. Notably, the model's accuracy improved as the experimental measurements became more consistent, making it a valuable tool for refining fabrication methods. This work represents the first application of an AI approach to the design of soft, non‐covalent assembled materials while addressing their intrinsic variability, demonstrating the potential of machine learning in this field. Beyond the specific system investigated here, the proposed GP‐based framework is amenable to being adapted to other soft and biofabricated materials, particularly those governed by multi‐parametric processes and intrinsic experimental variability.

## 4 Methods

### 4.1 Materials

Sodium alginate, sourced from brown algae (Mw = 120,000–190,000 g/mol), and dextran from *Leuconostoc spp*. (Mw = 450 000–650 000 Da), were purchased from Sigma‐Aldrich and used to prepare the Phase I solution. For the Phase II solution, poly(ethylene glycol) (Mw = 8,000 Da) from Sigma‐Aldrich and 𝜖‐poly‐L‐lysine (Epolyly Pure, Mw∼4,700 g/mol) produced via fermentation of *Streptomyces albulus* PD‐1, provided by Handary S.A. (Brussels, Belgium), were utilized. PBS pellets, also purchased from Sigma‐Aldrich, were used for the washing steps. Fluorescein 5(6)‐isothiocyanate (purity ≥90%, HPLC) from Sigma‐Aldrich was employed for fiber staining, while Fluorescein isothiocyanate‐dextran (Mw = 2,000 kDa), also from Sigma‐Aldrich, was used in the permeability assays.

### 4.2 Preparation of Tubular Core–Shell Biomaterials

Hollow structures were fabricated by electrostatic complexation of two oppositely charged polyelectrolytes at the interface of an aqueous two‐phase system. To prepare the system, 15 wt.% dextran (DEX) and 17 wt.% poly(ethylene glycol) (PEG) solutions were dissolved in PBS. Sodium alginate (ALG) was dissolved in the DEX solution (Phase I), while 𝜖‐poly‐L‐lysine (EPL) was mixed in the PEG solution (Phase II). The pH of Phase I was adjusted to 7.2–7.4, and for Phase II, solutions with pH 6.5 and 7.2 were used in separate experiments. The phases were mixed using a BioX bioprinter, which controlled the movement of the printer cartridge. Phase II served as the bath, into which Phase I was immersed by moving the needle in thread‐like motions using computer‐aided design (CAD) models made in Tinkercad, with the syringe movements programmed accordingly. Needle nozzles with sizes 21G, 25G, and 30G were used, with pressures set at 60 kPa, 90 kPa, and 180 kPa, and printing speeds of 13 mm/s, 10 mm/s, and 5 mm/s, respectively. After printing, long fibers were formed as a result of the polyelectrolyte complexation. Complexation times of 2, 5, and 10 min were applied for different samples. During the complexation, the long fibers were cut into smaller segments (∼1 cm in length) using a spatula. Once complexation was complete, the bath solution was removed and replaced with PBS to halt the reaction. Three washing steps were pursued in total, and then plates with samples were incubated for 24 h at 25°C and 37°C, separately. To investigate the effects of different parameters on the fabrication of tubular biomaterials, separate experimental conditions were tested. These included two incubation temperatures (25°C and 37°C), two pH levels of bath solution (6.5 and 7.2), three complexation times (2 min, 5 min, and 10 min), and three nozzle sizes (21G, 25G, and 30G). Each combination of these variables was prepared on a separate plate, with a total of total combinations = 2 × 2 × 3 × 3 = 36 combinations, enabling a thorough analysis of how these factors influenced the final structures.

### 4.3 Calculation of Stability Yield

After 24 h of incubation, the yield of fibers was evaluated for all the experimental conditions. From each plate containing 10 fibers, the number of stable samples and ruptured ones was recorded, and the yield was calculated using the equation below.

$$\mathrm{Yield}\;(\%)=\frac{\mathrm{Ns}}{\mathrm{Nt}}\;\times 100$$

where, *Yield* (%): Percentage of fibers that remained stable after 24 H; *Ns*: Number of fibers that were stable after 24 H of incubation; *Nt*: Total number of produced fibers.

### 4.4 Scanning Electron Microscopy Analysis

The morphology of the fibers was observed using scanning electron microscopy (SEM). Fibers were fabricated with the use of a 3D bioprinter. To dehydrate the samples, they were gradually immersed in ethanol solutions with increasing concentrations (30%, 50%, 70%, 80%, 90%, 96%, and 100% v/v), spending 15 min in each solution. Before dehydration, the edges of the fibers were cut transversely with a spatula to reveal their internal structure. To prepare the samples for SEM imaging, a thin layer of gold was applied to their surface through sputter coating for 3 min.

### 4.5 Characterization of Membrane Thickness

The membrane thickness was measured separately for each condition. The samples were stained with fluorescein isothiocyanate (FITC) for a confocal microscope (LSM 900, Carl Zeiss, Germany) equipped with the ZEN Imaging software. To prepare the fluorescein isothiocyanate (FITC) solution, 1 mg of FITC was dissolved in 1 mL of dimethyl sulfoxide (DMSO), resulting in a 0.1% w/v solution. Then, 10 µL of this solution was mixed with 1 mL of phosphate‐buffered saline (PBS). Three fibers were incubated in 1 mL of this FITC‐PBS solution at 4°C for 24 h. After incubation, the fibers were washed three times with PBS and then observed under a confocal microscope, where z‐stack images were captured. Using ImageJ, the 3D structures were analysed to measure the membrane thickness.

### 4.6 Characterization of Membrane Opacity

The membrane opacity for each condition was analysed after 24 h of fiber formation. Images of fibers were captured using a Primostar microscope (Zeiss) under the same light conditions in all experiments. To qualify the membrane darkening, the mean grey value was measured using ImageJ.

### 4.7 Calculation of Swelling Ratio

Swelling of the materials starts after the PBS washing steps. To calculate the swelling ratio, the width of the fibers was measured before washing and then at specific time points: immediately after washing (0 min) and at 10 min, 30 min, 60 min, and 1440 min. The swelling ratio was calculated using the equation shown below.

$$\mathrm{Swelling}\;\mathrm{Ratio}\;(\%)=\frac{\mathrm{Wt}-W0}{W0}\times 100$$

where, *Swelling Ratio (*%): The percentage increase in the material's width due to swelling; *Wt*: The fiber width at a specific time point; *W0*: The initial fiber width before washing

### 4.8 Determination of Membrane Porosity and Pore Size

The porosity and pore size of the membrane of the fibers were measured using a confocal microscope (LSM 900, Carl Zeiss, Germany) with ZEN Imaging software. To prepare the samples for imaging, they were stained with FITC. A FITC solution was prepared by dissolving 1 mg of FITC in 1 mL of dimethyl sulfoxide (DMSO), resulting in a 0.1% w/v concentration. Then, 10 µL of this solution was diluted in 1 mL of PBS. Three fibers were incubated in 1 mL of the FITC‐PBS solution at 4°C for 24 h. After incubation, the fibers were washed three times with PBS and imaged using Z‐stack settings on the confocal microscope. The 3D structures captured were analysed in ImageJ to determine the membrane's porosity and pore size.

### 4.9 Permeability Studies

Permeability assays were pursued using fluorescein isothiocyanate‐dextran (FITC‐dextran) with a molecular weight of 2000 kDa. FITC‐dextran was dissolved in Phase I, and fibers were fabricated as previously described, with the merging of the two phases conducted manually using a syringe. During the polyelectrolyte complexation time, the fibers were cut into 1 cm lengths, followed by washing steps using 10 mL of PBS per step. Fluorescent images of the fibers were captured at specific time points of 0 min, 10 min, 30 min, 60 min, and 120 min using a Fluorescence microscope (Axio Imager M2, Carl Zeiss, Germany). Ruptured fibers were excluded from the experiment. The fluorescence intensity of each fiber at each time point was measured using ImageJ. To standardize the data, the fluorescence intensity was normalized to the intensity measured after the first washing step, as this step had the highest release of molecules. The effects of various parameters on the permeability of the tubular biomaterials were assessed. These parameters included two incubation temperatures (25°C and 37°C), two bath solution pHs (6.5 and 7.2), two complexation times (2 min and 10 min), and two nozzle sizes (21G and 25G). Each combination of these variables was prepared on separate plates, resulting in a total of 16 experimental conditions (2 × 2 × 2 × 2 = 16).

The release of FITC‐dextran was monitored over a 2‐h period by measuring fluorescence intensity at 0 min, 10 min, 30 min, 60 min, and 120 min. Normalized fluorescence values were plotted as a function of time for each sample using GraphPad Prism 8. To assess release kinetics, the rate of change in fluorescence over time was estimated by fitting a linear regression to the time‐course data for each replicate. The resulting slope values were used as a comparative measure of release rate across different conditions. This approach provided a simplified means of quantifying differences in release profiles between samples.

### 4.10 Machine Learning Analysis

All GP‐related calculations were performed using the Python packages GPFlow (V2.9.2) [24] and TensorFlow (V2.12.1) [25, 26], relying on code adapted from previous work. Following the literature [27], a null mean function was used, µ_*i*_(*x_i_*)  =  0 and the radial basis function (RBF) was selected as the covariance function (also known as kernel). The RBF kernel is one of the simplest and most popular kernels in the literature, and was selected in this work to perform all GP‐related regression and predictions. A more detailed kernel sweep was not performed to avoid introducing extra hyperparameters and, thus, overfitting to the relatively small datasets studied. To assess experimental uncertainty, a trainable Gaussian likelihood was employed. Normalization of the features and labels was performed, being particularly important due to the zero‐mean function assumed for the labels. The employed normalization techniques included standardization, log‐standardization, and min‐max normalization. Further details can be found in Section S1.

## Funding

Fundação para a Ciência e a Tecnologia PTDC/BTM‐ORG/3215/2020; H2020 Marie Skłodowska‐Curie Actions 101073404.

## Conflicts of Interest

The authors declare no conflict of interest.

## Supporting information




**Supporting File**: advs74404‐sup‐0001‐SuppMat.pdf.

## Data Availability

The data that support the findings of this study are openly available in GPs_Biomaterials at https://github.com/dinisAbranches/GPs_Biomaterials, reference number 0.
