# Supplementary material for: Understanding Fabrication Variability in Core‐Shell Soft Biomaterials Using Stochastic Artificial Intelligence
Source: Adv Sci (Weinh). 2026 Apr 1;13(24):e22389. doi: 10.1002/advs.202522389 (PMC13116341; doi:10.1002/advs.202522389)
Supplement: Supplementary file 1 — Supporting File: advs74404‐sup‐0001‐SuppMat.pdf. [file ADVS-13-e22389-s001.pdf]

Supporting Information

Understanding Fabrication Variability in Core–Shell Soft Biomaterials Using Stochastic Artificial Intelligence

Maria Alexaki[1+], Lília M. S. Dias[2-4+], Raquel Gonçalves[1], Dinis O. Abranches[1*], Albano N. Carneiro Neto[2], Rute A. S. Ferreira[2*], Paulo S. B. André[3], João F. Mano[1*], Mariana B. Oliveira[1*]

[1] *CICECO—Aveiro Institute of Materials, Department of Chemistry, University of Aveiro, 3810-193 Aveiro, Portugal*

[2]*Department of Physics and CICECO—Aveiro Institute of Materials, University of Aveiro, 3810-193 Aveiro, Portugal*

[3]*Department of Electrical and Computer Engineering and Instituto de Telecomunicações, Instituto Superior Técnico, Universidade de Lisboa, 1049-001 Lisbon, Portugal*

[4]*Department of Electrical and Computer Engineering, Carnegie Mellon University, 15213, Pittsburgh, Pennsylvania, USA*

**Corresponding authors*

[+]Equal author contribution

S1. Additional Material Characterization

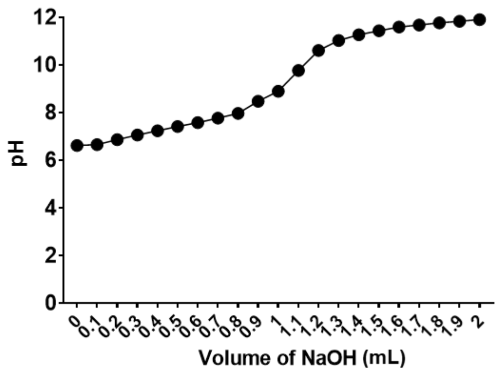

Figure S1. Titration curve of EPL with increasing volumes of 1M NaOH.

S2. Gaussian Process Analysis

GPs consider the training data as a multivariate normal probability distribution:[1,2]

$$\begin{bmatrix} Y_1 \\ \vdots \\ Y_N \end{bmatrix} \sim \mathcal{N}\left(\begin{bmatrix} \mu_1(\boldsymbol{x_1}) \\ \vdots \\ \mu_N(\boldsymbol{x_N}) \end{bmatrix}, \begin{bmatrix} k_{11}(\boldsymbol{x_1}, \boldsymbol{x_1}) & \cdots & k_{1N}(\boldsymbol{x_1}, \boldsymbol{x_N}) \\ \vdots & \ddots & \vdots \\ k_{N1}(\boldsymbol{x_N}, \boldsymbol{x_1}) & \cdots & k_{NN}(\boldsymbol{x_N}, \boldsymbol{x_N}) \end{bmatrix}\right) \tag{S1}$$

where N is the total number of training data points, $(\boldsymbol{x_i}, Y_i)$ represents training data point i, with $\boldsymbol{x_i}$ being the vector of features and Y_i the scalar label, μ_i is the i-th entry of the mean vector, and k_{ij} is the covariance between points i and j (as calculated using the kernel).

The radial basis function (RBF) was selected as the employed kernel, being defined as:

$$k_{ij}(\boldsymbol{x_i}, \boldsymbol{x_j}) = \sigma^2 \exp\left(-\frac{r(x_i, x_j)^2}{2l^2}\right) \tag{S2}$$

where $r(\boldsymbol{x_i}, \boldsymbol{x_j})$ is the Euclidian distance between points $\boldsymbol{x_i}$ and $\boldsymbol{x_j}$, and σ^2 and l are hyperparameters known as kernel variance and kernel length scale.[3]

Features and labels were normalized in this work using standardization (Equation S3), log-standardization (Equation S4) or Min-Max (Equation S5):

$$Y' = \frac{Y - \langle Y \rangle}{s_Y} \tag{S3}$$

$$Y' = \frac{\ln(Y) - \langle \ln(Y) \rangle}{s_{\ln(Y)}} \quad \text{(S4)}$$

$$x' = \frac{x - x_{min}}{x_{max} - x_{min}} \quad \text{(S5)}$$

where Y' is the normalized version of Y, and $\langle Y \rangle$ and s_Y represent its mean and standard deviation, respectively, and x_{min} and x_{max} are the minimum and maximum values of feature x, respectively [4].

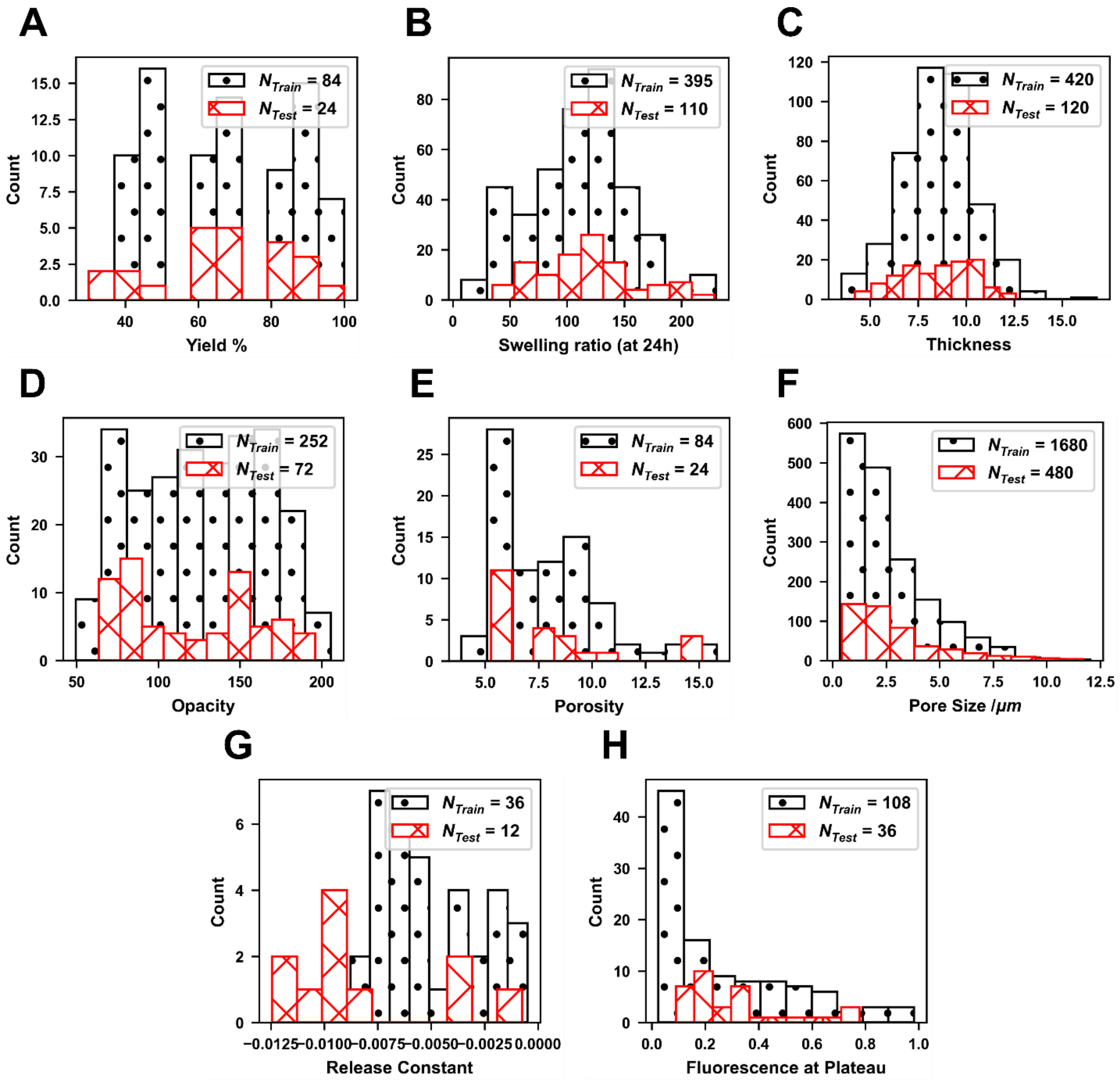

Figure S2. Count histograms of the training (black circles) and testing (red crosses) sets for the biomaterial properties measured in this work, namely yield of stable membranes obtained after washing (A), swelling ratio (B), thickness (C), opacity (D), porosity (E), pore size (F), release constant (G), and fluorescence at plateau (H). Each replicate measurement is considered as an independent data point.

Table S1. Feature and label normalization techniques employed in this work to train GP models for each property dataset, along with its range. (*adimensional)

Output	Feature norm.	Label norm.	Label Range [min, max]
1 - Yield of stable fiber formation (%)	None	Standardization	[30.0,100.0]
2 - Swelling ratio (at 24h) (%)	None	Standardization	[7.5, 230.0]
3 – Membrane thickness (μm)	None	Standardization	[3.5, 16.8]
4 – Membrane opacity*	None	Standardization	[49.6, 205.6]
5 – Porosity (%)	None	LogStand	[3.9,15.8]
6 - Pore Size (μm)	None	LogStand	[0.3,12.0]
7 - Release constant*	MinMax	Standardization	[-1.2×10^{-2} ,$- 4.9 \times 10^{-4}$]
8 – Relative fiber fluorescence at plateau*	MinMax	LogStand	[2.5×10^{-2}, 1.0]

References

1. Rasmussen, C. E. & Williams, C. K. I. Gaussian Processes for Machine Learning. *Gaussian Processes for Machine Learning* https://doi.org/10.7551/MITPRESS/3206.001.0001 (2005) doi:10.7551/MITPRESS/3206.001.0001.

2. Rasmussen, C. E. Gaussian Processes in Machine Learning. *Lecture Notes in Computer Science (including subseries Lecture Notes in Artificial Intelligence and Lecture Notes in Bioinformatics)* 3176, 63–71 (2004).

3. Matthews, A. G. D. G. *et al.* GPflow: A Gaussian process library using TensorFlow. *Journal of Machine Learning Research* 18, (2016).

4. Oliveria, J. D. Thermodynamics-Informed Machine Learning for the Design of Sustainable Materials: The Dawn of Digital Molecular Discovery. (University of Notre Dame, 2024). doi:https://doi.org/10.7274/25545994.
